# Real-world clinical outcomes of nivolumab and taxane as a second- or later-line therapy for recurrent or unresectable advanced esophageal squamous cell carcinoma

**DOI:** 10.3389/fonc.2023.1126536

**Published:** 2023-04-17

**Authors:** Manato Ohsawa, Yoichi Hamai, Manabu Emi, Yuta Ibuki, Tomoaki Kurokawa, Toru Yoshikawa, Ryosuke Hirohata, Nao Kitasaki, Morihito Okada

**Affiliations:** Department of Surgical Oncology, Research Institute for Radiation Biology and Medicine, Hiroshima University, Hiroshima, Japan

**Keywords:** esophageal squamous cell carcinoma, chemotherapy, immunotherapy, nivolumab, taxane

## Abstract

**Background:**

Nivolumab is approved in Japan as a second-line treatment for patients with advanced esophageal squamous cell carcinoma (ESCC) resistant to fluoropyrimidine and platinum-based drugs. It is also used in adjuvant and primary postoperative therapies. This study aimed to report real-world data on nivolumab use for esophageal cancer treatment.

**Methods:**

In total, 171 patients with recurrent or unresectable advanced ESCC who received nivolumab (n = 61) or taxane (n = 110) were included. We collected real-world data of patients treated with nivolumab as a second- or later-line therapy and evaluated treatment outcomes and safety.

**Results:**

Median overall survival was longer and progression-free survival (PFS) was significantly longer (p = 0.0172) in patients who received nivolumab than in patients who received taxane as a second- or later-line therapy. Furthermore, subgroup analysis for second-line treatment only showed the superiority of nivolumab in increasing the PFS rate (p = 0.0056). No serious adverse events were observed.

**Conclusions:**

In real-world practice, nivolumab was safer and more effective than taxane in patients with ESCC with diverse clinical profiles who did not meet trial eligibility criteria, including those with poor Eastern Cooperative Oncology Group performance status, comorbidities, and receiving multiple treatments.

## 1 Introduction

Squamous cell carcinoma is the most common histologic esophageal cancer type, accounting for approximately 90% of all cases worldwide (1, 2). In Japan, fluoropyrimidine plus platinum was used as a first-line treatment for unresectable, advanced, or recurrent esophageal cancer, and taxane-based drugs were used as a second-line treatment until the discovery of nivolumab (3, 4). Taxanes have hematological, gastrointestinal, and neurological adverse effects (5) and are associated with low long-term survival rates, rendering them less effective (6, 7).

Nivolumab is a fully human immunoglobulin-G4 monoclonal antibody that enhances T-cell anti-tumor activity by inhibiting the expression of the programmed cell death protein-1 (PD-1) receptor (8, 9). The efficacy and safety of nivolumab for the treatment of unresectable advanced or recurrent esophageal squamous cell carcinoma (ESCC) have been demonstrated in the ATTRACTION-1 trial, whereas the superiority of nivolumab over taxane has been demonstrated in the ATTRACTION-3 trial. Nivolumab has been approved as a new second-line treatment for patients with advanced ESCC who are resistant to fluoropyrimidine and platinum drugs (10, 11). The results of the CheckMate 577 and CheckMate 648 trials have demonstrated the efficacy of nivolumab as an adjuvant or first-line therapy (12, 13). However, data on the efficacy and adverse events (AEs) of nivolumab monotherapy in clinical practice are limited.

In clinical practice, nivolumab may also be used in patients who do not meet the eligibility criteria for clinical trials, including patients with poor Eastern Cooperative Oncology Group performance status (ECOG PS), those with comorbidities, and those receiving multiple treatments. Drug efficacy should be assessed in clinical trials and real-world settings. Real-world clinical data on nivolumab use have been reported for gastric and head and neck cancers (14, 15); however, the corresponding data for esophageal cancer have not been reported. Combination chemotherapy with nivolumab is being increasingly used in clinical settings; therefore, the availability of prospective data for nivolumab monotherapy is limited. Herein, we report real-world data on safety and outcomes in patients treated with nivolumab as a second- or later-line monotherapy, as well as in patients previously treated with taxanes as a second- or later-line treatment.

## 2 Methods

### 2.1 Patients

The study involved 171 patients with recurrent or unresectable advanced ESCC treated with nivolumab or taxane as a second- or later-line therapy at Hiroshima University Hospital from October 2008 to November 2021. Taxane was used in 110 patients from 2008 to 2020, and nivolumab was used in 61 patients from 2016 to 2021. Data on the clinical characteristics of patients were obtained from our surgical database and medical records. The clinicopathologic diagnosis of tumors was based on the tumor-lymph node-metastasis (TNM) classification (16). Clinical tumor response to nivolumab or taxane was evaluated according to the Response Evaluation Criteria in Solid Tumors (17). Five patients without target lesions (second-line therapy: two patients, later-line therapy: three patients) were identified and excluded from the analysis of treatment response and progression-free survival (PFS).

### 2.2 Treatment protocol

Nivolumab (240 mg) was administered intravenously for 30 min every two weeks (each cycle of six weeks). Paclitaxel (100 mg/m^2^) was administered for 60 min once weekly for six weeks, followed by no treatment for one week (each cycle of seven weeks). Docetaxel (75 mg/m^2^) was administered for 60 min every three weeks (each cycle of three weeks) until disease progression or toxicity was observed. No prophylaxis was used in the nivolumab group. In the paclitaxel and docetaxel groups, dexamethasone at 6.6 mg was administered as an antiemetic.

Treatment was interrupted or delayed in some patients owing to AEs. In such cases, the treatment was resumed when considered safe by the attending physician based on the patient’s general condition, symptoms, and blood test results; doses were reduced according to paclitaxel- and docetaxel-related toxicities. The dose was not reduced in patients administered nivolumab.

The results of the ATTRACTION-1 and ATTRACTION-3 trials showed that nivolumab is effective, leading to its approval for use in Japan regardless of programmed cell death protein-1 ligand-1 (PD-L1) expression (10, 11). Consequently, to reduce the economic burden on patients, we did not evaluate PD-L1 status as a part of routine practice in our institute. AEs were assessed according to the National Cancer Institute Common Terminology Criteria for Adverse Events (CTCAE) version 4.0. The data on AEs were obtained from our surgical database and were confirmed by reviewing the medical records and blood test results again. Patients who were already using oral medications for hormonal abnormalities prior to the start of treatment (one female patient with hypothyroidism and one female patient with hyperthyroidism) were not included in the analysis due to treatment-related AEs. When an AE occurred, basically at the point when the patient was rated grade 2 by the CTCAE, treatment by drug therapy, i.e., drugs to relieve symptoms, was used. For example, for diarrhea, bowel regulators and antidiarrheals were used.

The treatment plan for each patient with unresectable, advanced, or recurrent ESCC was defined after a discussion among the surgeons, oncologists, and radiologists. Blood tests, chest x-rays, electrocardiograms, echocardiograms, and pulmonary function tests were performed to evaluate the functional parameters of vital organs before treatment. Appropriate anti-tumor treatment approaches, such as chemotherapy, radiation therapy, surgery, or combinations of these modalities were recommended based on the patient’s overall condition, neoadjuvant or adjuvant therapy use, and metastasis site. Surgical resection was performed only in cases of solitary or localized recurrence and completely resectable tumors, such as in cases of localized lymph node recurrence or oligometastasis of the lung or skin. Symptomatic brain metastases were also surgically resected.

All patients underwent clinical response assessment using computed tomography (CT) imaging after three courses each of chemotherapy and nivolumab. The patients were also examined whenever their symptoms worsened. CT imaging was performed promptly for efficacy evaluation if the tumor volume was large, or the patient’s condition was poor. In contrast, if the patient’s condition was stable and some tumor shrinkage was observed, the examination was delayed at the discretion of the attending physician.

### 2.3 Statistical analysis

The results are presented as number (%) or median value unless stated otherwise. Comparisons between groups were performed using independent sample *t*-tests. Enumerated data were analyzed using a chi-squared (χ2) test. Survival rates were analyzed using the Kaplan–Meier curves and compared using the log-rank test. PFS was defined as the time from the date of nivolumab or taxane treatment initiation to the time when disease progression was determined. Overall survival (OS) was defined as the time from the date of nivolumab or taxane treatment initiation to death from any cause or the last follow-up visit. Patients who were admitted after May 2021 (nine patients in the nivolumab group) were excluded from the prognostic analysis owing to a short follow-up period. Statistical analyses were performed using JMP Pro 15 software (2019; SAS Institute, Cary, NC, USA). Statistical significance was set at p < 0.05.

### 2.4 Ethics statement

The Institutional Review Board of Hiroshima University (approval number: 2225) approved the study protocol and waived the need for informed consent from patients owing to the retrospective nature of the study.

## 3 Results

### 3.1 Clinicopathological characteristics

The clinicopathological characteristics of the 171 patients with recurrent or unresectable advanced ESCC (mean age, 66.2 ± 9.2 years; male, n = 151; female, n = 20) were compared between the nivolumab (n = 61) and taxane (n = 110) groups (**Table 1**).

**Table 1:** Clinicopathological characteristics of patients treated with nivolumab or taxane.

Parameter	n = 171	Nivolumab group (n = 61)	Taxane group (n = 110)	P
Age (mean ± SD, y)	66.2 ± 9.2	70.0 ± 8.3	64.1 ± 9.0	<0.0001
Sex				
Male	151 (88.3%)	52 (85.2%)	99 (90.0%)	0.8590
Female	20 (11.7%)	9 (14.8%)	11 (10.0%)	
ECOG PS				
0	94 (55.0%)	30 (49.2%)	64 (58.2%)	0.0503
1	69 (40.3%)	25 (41.0%)	44 (40.0%)	
2	8 (4.7%)	6 (9.8%)	2 (1.8%)	
History of smoking				
Never	21 (12.3%)	8 (13.1%)	13 (11.8%)	0.3396
Former	96 (56.1%)	38 (62.3%)	58 (52.7%)	
Current	54 (31.6%)	15 (24.6%)	39 (35.5%)	
Tumor makers				
SCC (mean ± SD, ng/mL)	3.9 ± 7.4	4.2 ± 8.5	3.7 ± 6.7	0.6729
CEA (mean ± SD, ng/mL)	9.1 ± 50.2	16.0 ± 82.9	5.3 ± 10.3	0.1807
Primary tumor location				
Cervical	24 (14.0%)	13 (21.3%)	11 (10.0%)	0.1021
Upper	27 (15.8%)	6 (9.8%)	21 (19.1%)	
Middle	75 (43.9%)	28 (45.9%)	47 (42.7%)	
Lower	45 (26.3%)	14 (23.0%)	31 (28.2%)	
Clinical T^a^				
cT1	16 (9.4%)	7 (11.5%)	9 (8.3%)	0.2858
cT2	9 (5.3%)	1 (1.6%)	8 (7.3%)	
cT3	113 (66.5%)	39 (63.9%)	74 (67.9%)	
cT4	32 (18.8%)	14 (23.0%)	18 (16.5%)	
Clinical N^a^				
cN0	28 (16.4%)	13 (21.3%)	15 (13.6%)	0.1042
cN1	59 (34.5%)	22 (36.1%)	37 (33.7%)	
cN2	51 (29.8%)	20 (32.8%)	31 (28.2%)	
cN3	33 (19.3%)	6 (9.8%)	27 (24.5%)	
Clinical M^a^				
cM0	103 (60.6%)	37 (60.7%)	67 (60.9%)	0.9077
cM1	67 (39.4%)	24 (39.3%)	43 (39.1%)	
Clinical stage^a^				
I	11 (6.4%)	6 (9.8%)	5 (4.5%)	0.3831
II	14 (8.2%)	6 (9.8%)	8 (7.3%)	
III	61 (35.7%)	18 (29.6%)	43 (39.1%)	
IV	85 (49.7%)	31 (50.8%)	54 (49.1%)	
Disease status				
Postoperative recurrence	79 (46.2%)	24 (39.3%)	55 (50.0%)	0.1806
No esophagectomy	92 (53.8%)	37 (60.7%)	55 (50.0%)	
Previous therapies^b^				
Surgery	79 (46.2%)	24 (39.3%)	55 (50.0%)	0.1806
Radiation therapy	131 (76.6%)	44 (72.1%)	87 (79.1%)	0.3031
Systemic anticancer therapy	171 (100%)	61 (100%)	110 (100%)	1.0000
Number of previous chemotherapies				
1	147 (86.0%)	48 (78.7%)	99 (90.0%)	0.2207
2	17 (9.9%)	9 (14.8%)	8 (7.2%)	
> 3	7 (4.1%)	4 (6.5%)	3 (2.8%)	
Histology of biopsy^c^				
Well-differentiated	7 (4.1%)	1 (1.6%)	6 (5.5%)	0.2370
Moderately differentiated	51 (29.8%)	20 (32.8%)	31 (28.2%)	
Poorly differentiated	51 (29.8%)	14 (23.0%)	37 (33.6%)	
Squamous cell carcinoma (not assessable)	62 (36.3%)	26 (42.6%)	36 (32.7%)	
Histology of surgical specimens^d^ (n = 79)				
Well-differentiated	5 (6.3%)	1 (4.2%)	4 (7.3%)	0.1002
Moderately differentiated	32 (40.5%)	11 (45.8%)	21 (38.2%)	
Poorly differentiated	29 (36.7%)	5 (20.8%)	24 (43.6%)	
Squamous cell carcinoma (not assessable)	13 (16.5%)	7 (29.2%)	6 (10.9%)	

SD, standard deviation; values are shown as n (%) or as mean ± SD; ECOG PS, Eastern Cooperative Oncology Group performance status; SCC, squamous cell carcinoma-related antigen; CEA,carcinoembryonic antigen. ^a^Pretherapeutic staging according to TNM classification, 8th edition. ^b^Some cases underwent more than one therapy before nivolumab or taxane therapy. ^c^Biopsy tissue from upper gastrointestinal endoscopy at the initial examination in all cases. ^d^Permanent pathology in surgical cases.

In contrast to clinical trials, this study involved cases with ECOG PS 2: 6 (9.8%) in the nivolumab group and 2 (1.8%) in the taxane group. Before the administration of nivolumab or taxane, 79 (46.2%), 131 (76.6%), and 171 (100%) patients were treated with surgery, radiation therapy, and chemotherapy, respectively. Patients in the nivolumab group were older than those in the taxane group (70.0 ± 8.3 versus 64.1 ± 9.0 years; p < 0.0001).

### 3.2 Effects of nivolumab and taxane

The effects of nivolumab and taxane are shown in **Table 2**. An objective response was defined as a complete or partial response. Disease control was defined as a complete response, partial response, or stable disease. As a second- or later-line therapy, nivolumab versus taxane yielded a complete response rate of 1 (1.6%) versus 3 (2.9%), an objective response rate of 12 (19.6%) versus 19 (18.1%), and a disease control rate of 28 (45.9%) versus 44 (41.9%), respectively. When nivolumab versus taxane was used only as a second-line therapy, the complete response rate was 1 (2.7%) versus 1 (1.3%), the objective response rate was 11 (29.7%) versus 12 (15.4%), and the disease control rate was 21 (56.7%) versus 32 (41.0%), respectively.

**Table 2:** Treatment response to nivolumab or taxane.

**Patients treated with nivolumab or taxane as a second- or later-line therapy**		
	**Nivolumab group (n = 61)**	**Taxane group (n = 105)**
Best overall response		
Complete response	1 (1.6%)	3 (2.9%)
Partial response	11 (18.0%)	16 (15.2%)
Stable disease	16 (26.2%)	25 (23.8%)
Progressive disease	33 (54.1%)	61 (58.1%)
**Objective response**	12 (19.6%)	19 (18.1%)
**Disease control**	28 (45.9%)	44 (41.9%)
**Patients treated with nivolumab or taxane as second-line therapy**		
	**Nivolumab group (n = 37)**	**Taxane group (n = 78)**
Best overall response		
Complete response	1 (2.7%)	1 (1.3%)
Partial response	10 (27.0%)	11 (14.1%)
Stable disease	10 (27.0%)	20 (25.6%)
Progressive disease	16 (43.3%)	46 (59.0%)
**Objective response**	11 (29.7%)	12 (15.4%)
**Disease control**	21 (56.7%)	32 (41.0%)

According to Response Evaluation Criteria in Solid Tumors (RECIST). Five patients without target lesions were excluded (second-line: 2 patients, later-line: 3 patients).

### 3.3 Nivolumab- and taxane-related AEs

In the nivolumab group, the major events resulting from non-hematologic toxicity were rash, fatigue, decreased appetite, and diarrhea (**Table 3**). Some hematologic toxicities were observed. In the taxane group, non-hematologic toxicities included fatigue, decreased appetite, diarrhea, arthralgia, nausea, alopecia, stomatitis, peripheral sensory neuropathy, and pneumonia. The most common hematologic toxicity-related events were decreased white blood cell and neutrophil counts, anemia, and febrile neutropenia.

**Table 3:** Summary of treatment-related adverse events.

	Nivolumab group (n = 61)				Taxane group (n = 110)			
	Grade 1-2	Grade 3	Grade 4	Grade 5	Grade 1-2	Grade 3	Grade 4	Grade 5
**All events** ^a^	31 (50.8%)	8 (13.1%)	0	0	57 (51.8%)	50 (45.4%)	26 (23.6%)	2 (1.8%)
**Rash**	9 (14.7%)	0	0	0	5 (4.5%)	0	0	0
**Fatigue**	7 (11.4%)	3 (4.9%)	0	0	27 (24.6%)	5 (4.5%)	0	0
**Decreased appetite**	7 (11.4%)	3 (4.9%)	0	0	19 (17.3%)	5 (4.5%)	0	0
**Diarrhea**	6 (9.8%)	0	0	0	8 (7.2%)	1 (0.9%)	0	0
**Arthralgia**	3 (4.9%)	0	0	0	14 (12.7%)	0	0	0
**Nausea**	2 (3.3%)	1 (1.6%)	0	0	25 (22.7%)	0	0	0
**Vertigo**	1 (1.6%)	0	0	0	2 (1.8%)	0	0	0
**Edema**	1 (1.6%)	0	0	0	0	0	0	0
**Melena**	1 (1.6%)	0	0	0	0	0	0	0
**Alopecia**	0	0	0	0	49 (44.5%)	0	0	0
**Stomatitis**	0	0	0	0	15 (13.6%)	1 (0.9%)	0	0
**Paronychia**	0	0	0	0	1 (0.9%)	0	0	0
**Peripheral sensory neuropathy**	0	0	0	0	15 (13.6%)	0	0	0
**Interstitial pneumonia**	1 (1.6%)	1 (1.6%)	0	0	2 (1.8%)	1 (0.9%)	1 (0.9%)	1 (0.9%)
**Pneumonia**	4 (6.6%)	2 (3.3%)	0	0	7 (6.3%)	0	0	1 (0.9%)
**Pancreatitis**	2 (3.3%)	0	0	0	0	0	0	0
**White blood cell count decrease**	0	0	0	0	15 (13.6%)	25 (22.7%)	9 (8.1%)	0
**Neutrophil count decrease**	1 (1.6%)	0	0	0	8 (7.3%)	23 (20.9%)	22 (20.0%)	0
**Anemia**	1 (1.6%)	0	0	0	15 (13.6%)	10 (9.1%)	0	0
**Febrile neutropenia**	0	0	0	0	0	10 (9.1%)	4 (3.6%)	0
**Hepatopathy**	3 (4.9%)	0	0	0	0	0	0	0
**Renal dysfunction**	1 (1.6%)	0	0	0	0	0	0	0
**Adrenal hypofunction**	0	1 (1.6%)	0	0	0	0	0	0
**Thyroid hypofunction**	8 (13.1%)	0	0	0	0	0	0	0

According to the National Cancer Institute Common Terminology Criteria for Adverse Events (CTCAE), version 4.0. ^a^Number of patients who showed adverse events, categorized by Grade.

Severe treatment-related AEs were recorded in eight of the 61 (13.1%) patients in the nivolumab group (grade 3, 8 [13.1%]; grade 4, 0; grade 5, 0) and 78 of the 110 (70.8%) patients in the taxane group (grade 3, 50 [45.4%]; grade 4, 26 [23.6%]; grade 5, 2 [1.8%]). The treatment-related AEs that led to treatment discontinuation were interstitial pneumonia (n = 1 [1.6%]) in the nivolumab group and interstitial pneumonia (n = 3 [2.7%]) and pneumonia (n = 1 [0.9%]) in the taxane group. Dose delays and reductions due to treatment-related AEs were more common in the taxane group (n = 32 [29.0%]) than in the nivolumab group (n = 7 [11.5%]). Rash, interstitial pneumonia, pancreatitis, hepatopathy, renal dysfunction, adrenal hypofunction, and thyroid hypofunction were immune-related AEs and adverse events that were characteristic of nivolumab. Immune-related AEs were observed in 20 patients (32.7%) in the nivolumab group. The most common immune-related AEs were rash in 9 (14.7%) patients and thyroid hypofunction in 8 (13.1%) patients. A total of nine patients, all of whom were male, received hormones-affecting drugs as a therapy during this treatment. In the nivolumab group, four male patients who developed thyroid hypofunction were treated with levothyroxine sodium hydrate. One male patient with adrenal hypofunction was treated with hydrocortisone. One male patient with interstitial pneumonia was treated with prednisolone and methylprednisolone. In the taxane group, three male patients with interstitial pneumonia were treated with prednisolone and methylprednisolone.

### 3.4 OS and PFS of patients treated with nivolumab or taxane

**Figure 1:**
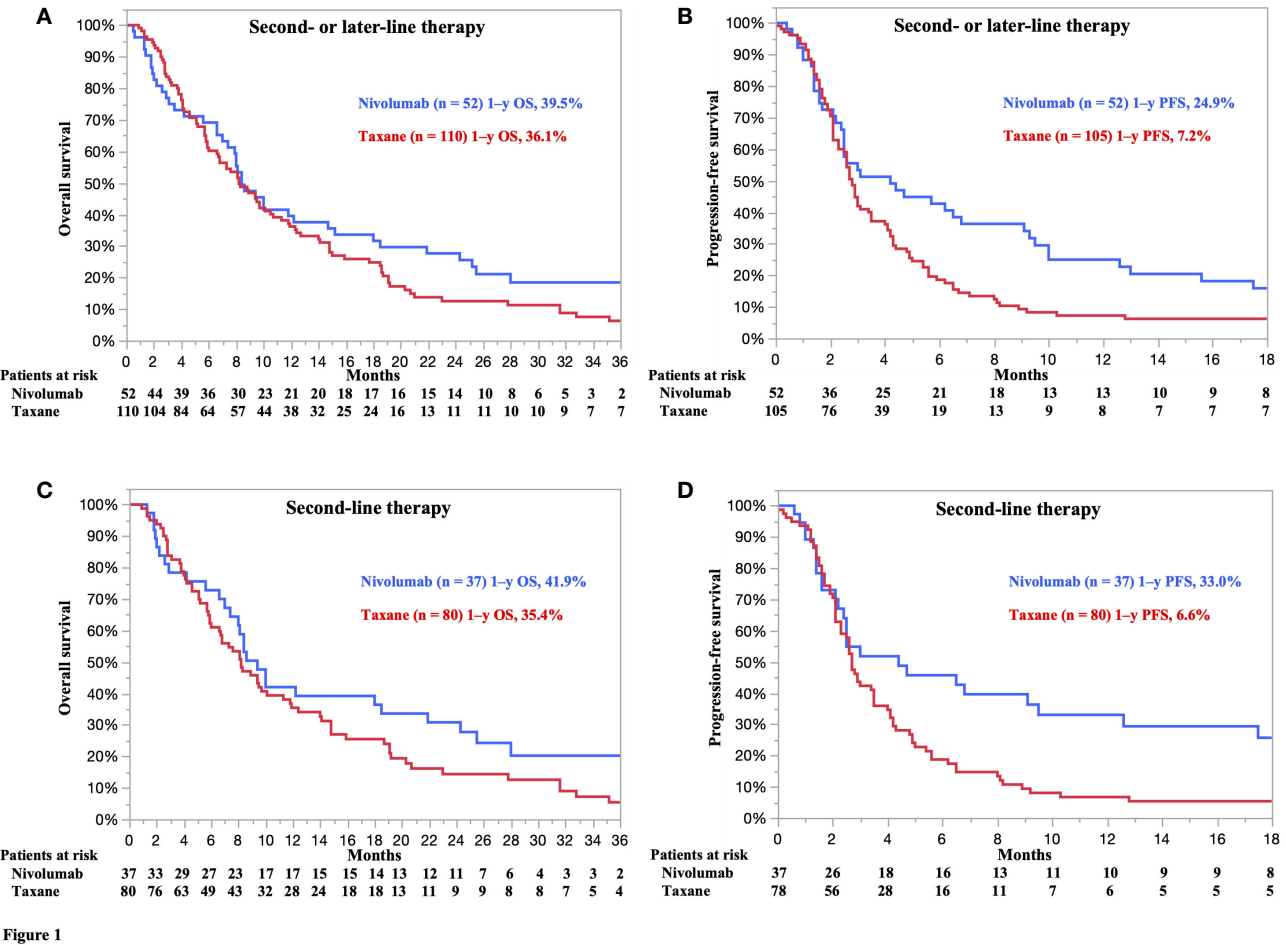
**(A)** Overall survival (OS) and **(B)** progression-free survival (PFS) of all patients treated with nivolumab or taxane as a second- or later-line treatment. **(C)** OS and **(D)** PFS of patients treated with nivolumab or taxane as a second-line treatment. Five patients without target lesions were excluded from the analysis of PFS (second-line therapy: 2 patients, later-line therapy: 3 patients). **(A)** Hazard ratio (HR) for death 0.82 [95% confidence interval (CI) 0.58–1.18]; p = 0.3023. Nivolumab, median 8.4 months (95% CI 7.4–14.7). Taxane, median 8.2 months (95% CI 6.6–10.5). **(B)** HR for death 0.64 (95% CI 0.44–0.92); p = 0.0172. Nivolumab, median 4.2 months (95% CI 2.5–6.8). Taxane, median 2.8 months (95% CI 2.5–3.4). **(C)** HR for death 0.77 (95% CI 0.49–1.19); p = 0.2455. Nivolumab, median 9.4 months (95% CI 7.0–18.5). Taxane, median 8.2 months (95% CI 6.0–10.1). **(D)** HR for death 0.53 (95% CI 0.34–0.83); p = 0.0056. Nivolumab, median 4.4 months (95% CI 2.2–9.5). Taxane, median 2.7 months (95% CI 2.3–3.5).

A prognostic analysis with a follow-up period of at least 15 months was performed for patients in both the nivolumab and taxane groups. The treatment-specific survival curves for nivolumab versus taxane as a second- or later-line treatment showed numerically better OS rates in the former than in the latter group (hazard ratio [HR] 0.82, 95% confidence interval [CI] 0.58–1.18, p = 0.3023) (**Figure 1A**). The median OS estimates of patients in the nivolumab and taxane groups were 8.4 (95% CI 7.4–14.7) and 8.2 months (95% CI 6.6–10.5), respectively. The 1-, 2-, and 3-year survival rates of patients in the nivolumab versus taxane groups were 39.5% versus 36.1%, 25.5% versus 12.4%, and 18.4% versus 6.4%, respectively.

The treatment-specific PFS curves for nivolumab versus taxane as a second- or later-line treatment showed statistically better PFS rates in the former than in the latter group (HR 0.64, 95% CI 0.44–0.92, p = 0.0172) (**Figure 1B**). The median PFS estimates of the nivolumab and taxane groups were 4.2 (95% CI 2.5–6.8) and 2.8 (95% CI 2.5–3.4), respectively. The 6- and 12-month PFS rates of the nivolumab versus taxane groups were 42.7% versus 18.5% and 24.9% versus 7.2%, respectively.

### 3.5 OS and PFS of patients treated with nivolumab or taxane as a second-line treatment

The treatment-specific survival curves for nivolumab versus taxane as a second-line treatment showed numerically better OS rates in the former than in the latter group (HR 0.77, 95% CI 0.49–1.19, p = 0.2455) (**Figure 1C**). The median OS rates of patients in the nivolumab and taxane groups were 9.4 (95% CI 7.0–18.5) and 8.2 months (95% CI 6.0–10.1), respectively. The 1-, 2-, and 3-year OS rates of patients in the nivolumab versus taxane groups were 41.9% versus 35.4%, 30.7% versus 14.3%, and 20.2% versus 5.4%, respectively.

The treatment-specific PFS curves for nivolumab versus taxane as a second-line treatment showed statistically better PFS outcomes in the former than in the latter group (HR 0.53, 95% CI 0.34–0.83, p = 0.0056) (**Figure 1D**). The median PFS estimates of the nivolumab and taxane groups were 4.4 (95% CI 2.2–9.5) and 2.7 (95% CI 2.3–3.5), respectively. The 6- and 12-month PFS rates of the nivolumab and taxane groups were 45.7% versus 18.7% and 33.0% versus 6.6%, respectively.

## 4 Discussion

Herein, we summarized real-world clinical data on the efficacy and safety of nivolumab monotherapy and taxane as a second- or later-line treatment. The present study findings may help elucidate real-world outcomes, including those in patients with diverse clinical profiles who do not meet trial eligibility criteria—patients with poor ECOG PS, patients with comorbidities, and patients receiving multiple treatments.

The proportion of Japanese patients in the ATTRACTION-3 trial was 65.4% (274/419). The efficacy and safety of nivolumab compared with those of taxane were reported by Takahashi et al. in a study with baseline characteristics of patients compared to those of patients in this study (18). However, the present study included fewer patients with ECOG PS 0 in the nivolumab group than the previous study (49.2% [30/61] vs. 61.0% [83/136]). The inclusion of patients with ECOG PS 2 is a unique feature of real-world datasets; patients with poor general health who do not qualify for clinical trials are administered treatment in clinical practice.

As a second-line treatment, nivolumab yielded better median OS rates than taxane (9.4 vs. 8.2 months). This finding is comparable to that of the ATTRACTION-3 trial, which reported an OS rate of 10.9 months and an OS rate of 13.4 months in a Japanese subpopulation (11, 18). The corresponding estimates for the taxane groups in the ATTRACTION-3 trial were 8.4 and 9.4 months, respectively (11, 18). The slightly poorer OS estimates in this study than in the previous study could be attributed to the discrepancies in study population characteristics; clinical trials have clearly defined enrolment criteria in contrast to clinical practice. The proportion of patients with ECOG PS 0 in the nivolumab group was lower than that in the taxane group; patients with poor ECOG PS or clinical status were more likely to be ineligible for the subsequent treatment. The higher mean age of the nivolumab group in this study than in previous reports and the fact that the nivolumab was administered to older patients may also have influenced the results (11, 18).

In the second-line treatment, the median PFS was significantly longer in the nivolumab group than in the taxane group (4.4 vs. 2.7 months). The corresponding values for nivolumab in the ATTRACTION-3 trial were 1.7 and 2.7 months in the overall population and Japanese subpopulation, respectively. In the taxane group, the corresponding values in the ATTRACTION-3 trial were 3.4 and 3.8 months, respectively, which were comparable to those in this study (11, 18). The superiority of nivolumab in this study over that in the ATTRACTION-3 trial may be due to several factors. First, in clinical trials, CT imaging evaluations are performed at strictly defined time points; in contrast, in clinical practice, CT imaging evaluations may be postponed in patients whose condition is stable and who have achieved a certain degree of tumor shrinkage.

In addition, clinical trials involve high rates of protocol adherence; in contrast, in clinical practice, the patient’s condition may preclude treatment completion every two weeks, as scheduled. The interval between imaging exams may be further extended if the patient’s general condition deteriorates to the point where the continuation of treatment becomes difficult and the patient is transferred for the best supportive care. Thus, the interval between imaging evaluations may have been slightly extended in the real-world dataset, increasing the intervals between PFS assessments.

The Kaplan–Meier curve obtained in this study was comparable to that obtained in the ATTRACTION-3 trial for the overall population and Japanese subpopulation. The curves crossed after approximately four months, with nivolumab being superior to taxane in terms of both OS and PFS (11, 18). These results reflect the characteristics of immune checkpoint inhibitors, which are ineffective in some patients and may lead to survival curve dips, as well as long-lasting effects in patients with a responsive disease. Factors associated with immune checkpoint inhibitor efficacy include PD-1 and PD-L1 expression, mutation burden, CD8 lymphocyte count, interferon-γ level, and interleukin 12; nevertheless, it remains challenging to accurately predict clinical efficacy (19–22). Recent meta-analysis reports on the association between PD-L1 and immune checkpoint inhibitor treatment efficacy have shown no survival benefit of immune checkpoint inhibitor-based regimens compared to chemotherapy alone in subgroups with tumor proportion scores of less than 1% (23). Further studies are required to identify predictors of treatment efficacy in this context.

The long-lasting efficacy of immune checkpoint inhibitors in responsive cases is referred to as a “tail plateau” (24). Our results showed a tail plateau in the nivolumab group; a similar pattern was not observed in the taxane group. This long-lasting effect may have resulted in the superiority of the nivolumab group over the taxane group in terms of OS and PFS. Although the detailed mechanisms underlying the tail plateaus are unknown, it is possible that an immunological memory, a key feature of the adaptive immune system, is responsible for this prolonged response. Specifically, the adaptive immune system can mount a sustained response to a specific epitope or antigen over an extended period (25, 26).

The safety results are comparable to those of the Japanese subpopulation in the ATTRACTION-3 trial, although there were more cases of grade 1 and 2 AEs in the taxane group (18). Immune-related AEs are discrete toxicities caused by nonspecific activation of the immune system that can affect almost any organ system. Several studies have reported AE rates of less than 30% for anti-PD-1 agents (27, 28). Here, the incidence of immune-related AEs was 20 (32.7%) in the nivolumab group.

Immune-related AEs may include cutaneous, gastrointestinal, endocrine, pulmonary, and musculoskeletal events, which are well-known and commonly experienced. Cardiac, hematologic, renal, neurologic, and ophthalmologic events are also well-known but not frequent (29). Most AEs are mild to moderate; however, severe and life-threatening AEs have been reported, with treatment-related mortality rates of up to 2% in clinical trials (27, 30). Here, there were no cases of treatment-related deaths. Immune-related AEs are rarely severe; however, the associated risks should be considered and identified early when using this treatment.

This study has some limitations. It was based on data from a single institution, and the number of patients was relatively small. Furthermore, the results of the ATTRACTION-3 trial showed that nivolumab was effective and thus, it was approved for use in patients with and without PD-L1 expression. Therefore, PD-L1 expression could not be evaluated. Many patients in the taxane group were treated before nivolumab was approved as a second-line treatment, and the timing of treatment in the two groups differed. Different treatment timings may have led to differences in treatment management. In addition, docetaxel and paclitaxel were examined collectively as a taxane group, similar to that in the ATTRACTION-3 trial, but the efficacy and safety of the two drugs may differ. Subjects whose drug dose was reduced according to their general condition were also included.

Unlike the ATTRACTION-3 trial, this study provides real-world data on patients with diverse profiles, such as those with poor ECOG PS, those with comorbidities, and those receiving multiple treatments. However, nivolumab monotherapy did not cause any serious AEs in this study. In addition, the patients treated with nivolumab had numerically longer OS and statistically longer PFS than patients treated with taxanes.

In conclusion, nivolumab was safer to use and more effective than taxane in real-world practice for patients with ESCC with diverse clinical profiles who did not meet trial eligibility criteria, including patients with poor ECOG PS, patients with comorbidities, and patients receiving multiple treatments.

## Data availability statement

The original contributions presented in the study are included in the article/supplementary material. Further inquiries can be directed to the corresponding author.

## Author contributions

MOh and YH drafted the manuscript. MOh, YH, ME, YI, TK, TY, RH, and NK contributed to patient care. MOh and YH performed the literature search. MOh, YH, ME, YI, TK, TY, RH, NK, and MOk critically revised the manuscript. All authors contributed to the article and approved the submitted version.
